# Supplementary material for: Acoustic Holographic Rendering with Two-dimensional Metamaterial-based Passive Phased Array
Source: Sci Rep. 2016 Oct 14;6:35437. doi: 10.1038/srep35437 (PMC5064310; doi:10.1038/srep35437)
Supplement: Supplementary Information [file srep35437-s1.pdf]

Supplementary Information

Acoustic Holographic Rendering with Two-dimensional Metamaterial-based Passive Phased Array

Yangbo Xie[1]*, Chen Shen[2]*, Wenqi Wang[1], Junfei Li[1], Dingjie Suo[2], Bogdan-Ioan Popa[1], Yun Jing[2††], and Steven A. Cummer[1†]

[1]Department of Electrical and Computer Engineering, Duke University, Durham, North Carolina 27708, USA

[2]Department of Mechanical and Aerospace Engineering, North Carolina State University, Raleigh, North Carolina 27695, USA

* These authors contributed equally to this work

Corresponding authors:
† cummer@ee.duke.edu
†† yjing2@ncsu.edu;

Supplementary Methods: GSW Hologram Generation Algorithm

Consider an array of acoustic sources with N elements, the sound pressure at the *m*-th target ($m = 1,\ 2,\ \ldots,\ M$) can be calculated by propagating each source element to it:

$$p_m = i\frac{\rho ckS}{2\pi}\sum_n a_n e^{i\varphi_n}\frac{e^{-ikd_{mn}}}{d_{mn}} \qquad (1)$$

Where a_n and φ_n are the amplitude and phase of the radiating velocity the *n*-th element on the source surface, respectively; d_{mn} is the distance between the *n*-th element and *m*-th target; ρ and c are the density and sound speed of the background medium, respectively; k is the wave number; S is the surface area of each element.

Expressing Equation (1) with matrix notation: $\mathbf{P} = \mathbf{HU}$, where $\mathbf{P}$ and $\mathbf{U}$ are the $M \times 1$ and $N \times 1$ pressure vectors at each target and source element, $\mathbf{H}$ is the transferring matrix given by

$$\mathbf{H}(m,n) = iK\frac{e^{-ikd_{mn}}}{d_{mn}} \qquad (2)$$

Where $K = \frac{\rho ckS}{2\pi}$ is considered to be constant since the source surface areas are identical.

Here we introduce the weighted Gerchberg-Saxton [2], or GSW algorithm to compute the optimal phase for each element, while the amplitude is assumed to be uniform on the source plane. We now throw away the constant term K and a_n, and the total radiation delivered to each target can be simplified as:

$$p_m = \sum_n \frac{e^{i(\varphi_n - kd_{mn})}}{d_{mn}} \qquad (3)$$

Next an iterative process is employed in order to minimize the non-uniformity of the amplitude of the non-zero elements on the projected image plane. A weighting coefficient w_m, thereby, is introduced for each target. The mean amplitude of all the non-zero elements on the image plane can be expressed as $\langle p \rangle = \frac{1}{M}\sum_m w_m \left| \sum_n \frac{e^{i(\varphi_n - kd_{mn})}}{d_{mn}} \right|$ and the iterative process is as follows:

Initial (0th) step: ${w_m}^0 = 1$;

1st step: ${w_m}^1 = \frac{\langle |p^0| \rangle}{|{p_m}^0|}$;

2nd step: $w_m{}^2 = w_m{}^1 \frac{\left\langle \left| p^1 \right| \right\rangle}{\left| p_m{}^1 \right|}$;

…

kth step: $w_m{}^k = w_m{}^{k-1} \frac{\left\langle \left| p^{k-1} \right| \right\rangle}{\left| p_m{}^{k-1} \right|}$

Eventually the optimal solution for the phase of each element can be calculated as:

$$\varphi_n = \arg\left\{ \sum_m w_m e^{ikd_{mn}} \frac{p_m}{\left| p_m \right|} \right\} \tag{4}$$

And the convergence criteria is set as $\frac{\min\{|p_1|,|p_2|,...,|p_m|\}}{\max\{|p_1|,|p_2|,...,|p_m|\}} < \delta^{stop}$.

Supplementary Methods: Angular Spectrum approach (ASA) of the Holographic Reconstructions

To facilitate the hologram design process and verify the GSW-based designs, Angular Spectrum approach[1] is employed. The complex field of the object plane (0 cm behind the hologram, $z = z_{obj}$) was decomposed into components with various spatial frequencies, and then propagated to the image plane ($z = z_{img}$ and 30 cm behind the hologram in our cases) by multiplying corresponding phase delay terms:

$$P(f_x, f_y; z_{img}) = P(f_x, f_y; z_{obj}) e^{j\sqrt{k_0{}^2 - f_x{}^2 - f_y{}^2}(z_{img} - z_{obj})} ,$$

where $P(f_x, f_y; z_{obj})$ is the Fourier component with spatial frequencies f_x and f_y in both dimensions on the object plane and $P(f_x, f_y; z_{img})$ is the projected Fourier component on the image plane. The complex field on the image plane can be reconstructed by performing an inverse Fourier Transform of all the Fourier components.

Supplementary Methods: Anechoic Chamber Measurement of the Holographic Reconstructions

The measurement was performed in an anechoic chamber to prevent the reverberation caused by the environment. The experimental setup is depicted in Fig. 2D, where a single speaker acting as

a point source emitted a Gaussian modulated sinusoidal pulse centered at 4 kHz towards the hologram. A scanning stage behind the hologram, with a microelectromechanical microphone (Analog Devices ADMP401) mounted on the bearing towards the hologram, measured the projected field on the image plane in a point-by-point fashion. The measurement at each position on the image plane was averaged over three times. Measured field patterns were then obtained from time domain measurements via Fourier transform.

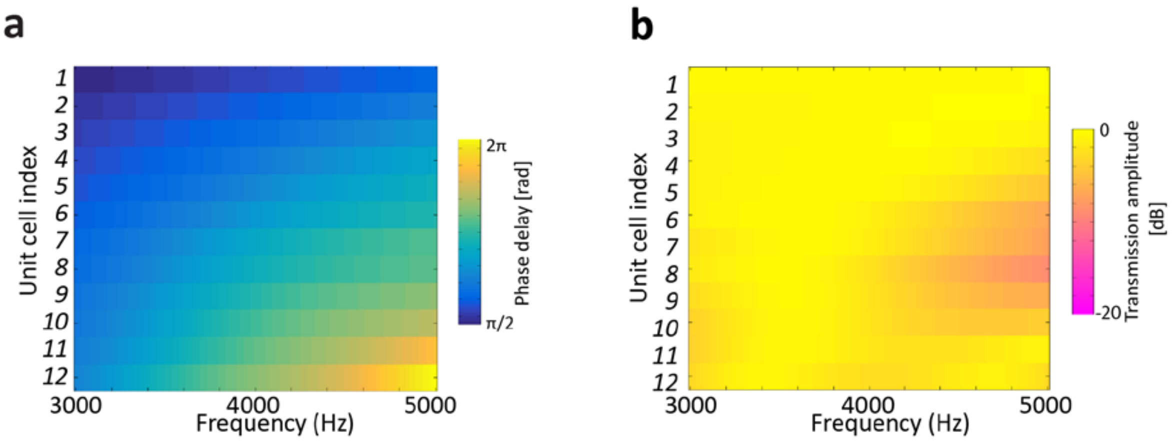

Fig. S1 | The characteristics of the set of 12 labyrinthine unit cells optimized for the hologram design. **a**, The retrieved transmission phase delays for the unit cells over the frequency span from 3 to 5 kHz. **b**, The retrieved transmission amplitudes for the unit cells over the frequency span from 3 to 5 kHz.

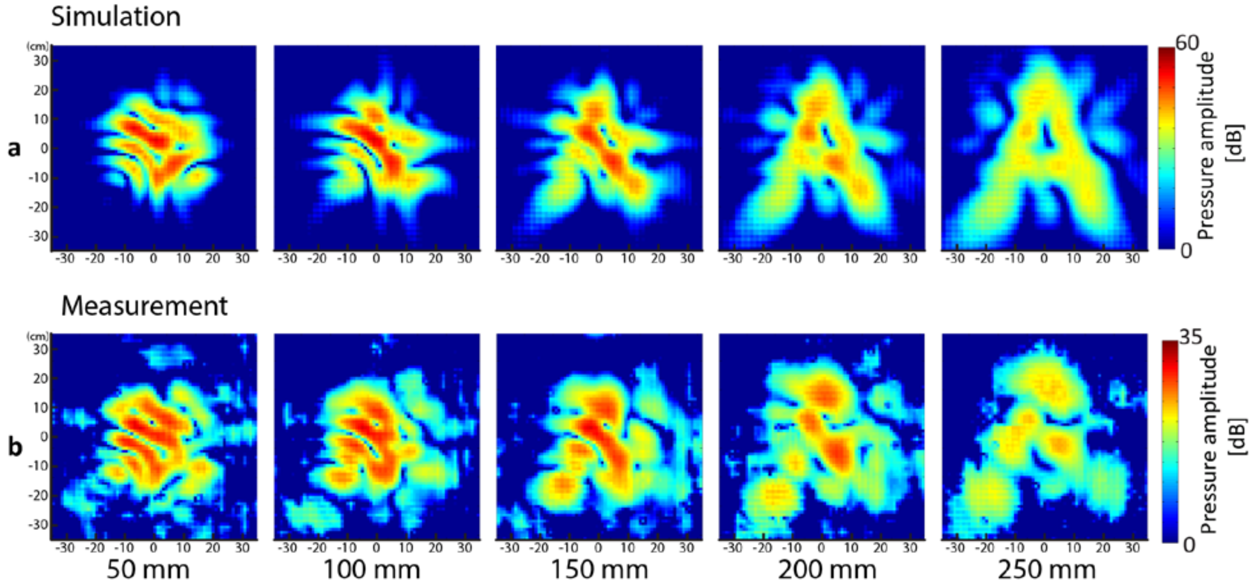

Fig. S2 | The simulated and measured field patterns (amplitude) at the depth from 50 mm to 250 mm, with 50 mm interval, for the hologram that projects an 'A'. **a**, The simulated field patterns obtained from Angular Spectrum calculation. **b**, The measured field patterns at the corresponding depths. Excellent agreement between the measurements and the simulations are observed at different depths. This series of measurement again verify the implementation of the hologram matches well with the design.

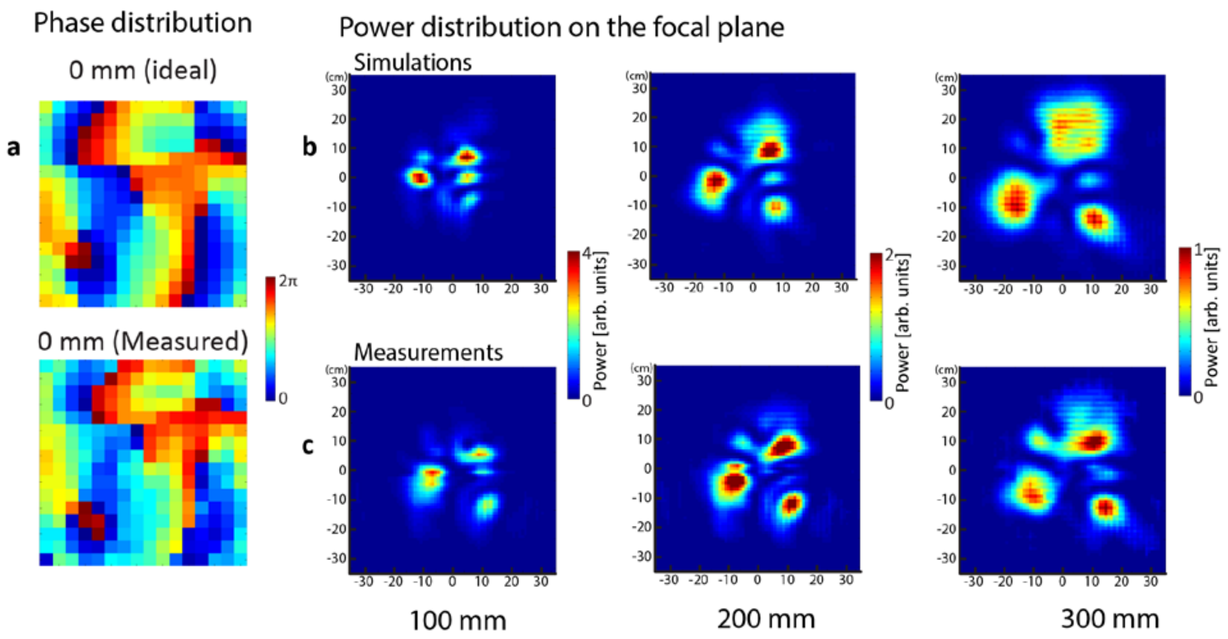

Fig. S3 | The simulated and measured phase distributions of the holographic lens and the field patterns (amplitude) at the depth from 100 mm to 300 mm, with 100 mm interval, for the multi-focal lensing. **a**, The simulated and measured phase distributions behind the holographic lens. **b**, The simulated field patterns obtained from Angular Spectrum calculation. **b**, The measured field patterns at the corresponding depths. Excellent agreement between the measurements and the simulations are also observed at these depths.

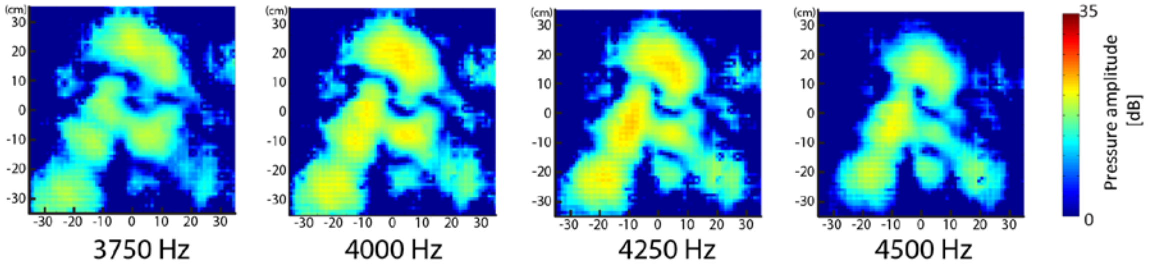

Fig. S4 | The measured field patterns at different frequencies on the designed reconstruction plane (300 mm) for the holographic reconstruction of the pattern ‘A’.

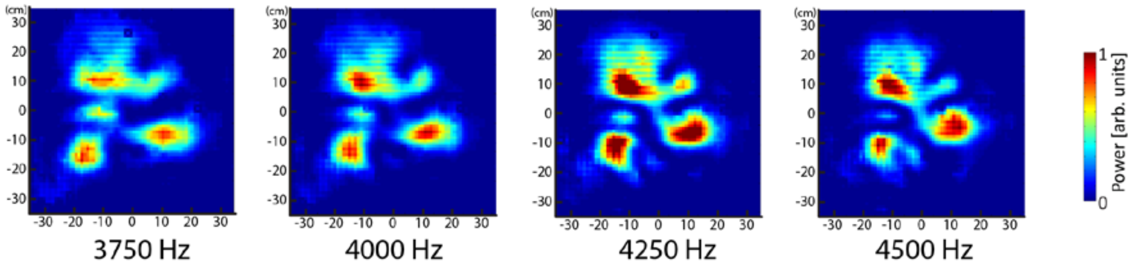

Fig. S5 | The measured field patterns at different frequencies on the designed reconstruction plane (300 mm) for the holographic lensing of three focal spots.

Supplementary References

1. William, E. G. *Fourier acoustics: sound radiation and nearfield acoustical holography* (Academic press, New York, 1999*)*
2. Hertzberg, Y., Naor, O., Volovick, A., & Shoham, S. Towards multifocal ultrasonic neural stimulation: pattern generation algorithms. *J. Neural. Eng.*, **7**, 056002 (2010).
